# Book Reviews

**Published:** 1917-02

**Authors:** 


### BOOK REVIEWS

---

Books mentioned may be inspected at and ordered through this office. So far as possible, books received in any month will be reviewed in the issue of the second month following. Pamphlets, quarterly and similar periodicals, reports, transactions, etc., will, as a rule, merely be mentioned.

---

**Diseases of the Nervous System.** By John Eastman Wilson, A. B., M. D., N. Y. Second edition. 682 pages, large 8 vo. Cloth, $6.00 net. Philadelphia: Boericke & Tafel, 1916.

Anatomic considerations are first presented, under the general title of "Architecture of the Nervous System." Physiology is discussed under the discussion of general symptoms, including the localization of lesions in the brain and cord. The classification adopted for the detailed discussion of diseases is mainly anatomic but some of the groupings necessarily depart from this method, as: progressive muscular atrophies and dystrophies; the apoplexies (including thrombosis and embolism); functional nervous diseases (epilepsy and hysteria); spasmodic diseases, etc. By the use of paragraph headings as of age, definition, etiology, prognosis, etc., both clearness and brevity are served. The author may be criticized for distinguishing between "treatment" and "therapy" and for using the latter term mainly in the sense of drugs but the distinction of meaning is practically well taken, especially as the drugs used, mentioned only in general terms, might in some instances be open to conflict of opinion. The book is comprehensive and scholarly.

---

**Encyclopedia of Foods and Beverages.** Compiled by Artemas Ward, 50 Union Square, N. Y., 748 pages, copiously illustrated, with engravings and colored plates, $10.

This is stated to be a special edition, under a more general title, of the Grocer's Encyclopedia; A Compendium of Useful Information Concerning Foods of all Kinds, how they are raised, prepared and marketed; how to care for them in the store and home; how best to use and enjoy them, etc. The present title gives a fairer idea of the scope of the work, rather because the original one implies a limitation of study and availability. The work is unique and difficult to describe in brief. It might be spoken of as the pharmacognosy of dietetics but, in addition, it includes a description of twine, paper, etc., included in grocers' stocks but not coming under the classification of foods. It is eminently practical and makes no pretense of covering scientific details of dietetics but it is by no means superficial, nor solely commercial. By a polyglot and unilingual glossary, the common food names are given for French, German, Italian and Swedish. There is an equally needed glossary of "culinary and bill-of-fare items." The colored, full page plates of food plants, fish, cheese, honey, etc., are really beautiful. Beginning where the standard text books on dietetics leave off, it gives a vast amount of practical and scientific information about foods, natural and artificial, which every one who has any serious interest in dietetics, food sanitation, economics of foods, etc., will find invaluable. It will save many hours of unavailing consultation of botanies, inquiries of horticulturists and other raisers of foods, and similar attempts to learn more about foods than is given in ordinary text books.

**The Sexual Disabilities of Man.** Arthur Cooper, London. Published by Paul B. Hoeber, 69 E. 59, N. Y. 3d edition, revised and enlarged, 227 pages, $2.50.

It should be understood that this work is devoted to impotence and sterility, rather than to venereal diseases, which are considered only as etiologic factors of the former conditions. Beginning with a very lucid consideration of the semen and zoosperms, it deals both theoretically and practically with the various phases of the subject, in a highly satisfactory manner.

---

**Homoeopathic Therapeutics in Ophthalmology.** By John L. Moffat, B. S., M. D., O. et A. Chir, Ithaca. 166 pages, cloth. $1.25 net. Postpaid. Philadelphia: Boericke & Tafel. 1916.

After a preliminary consideration of Homoeopathy in general, this work takes up the principle items in the materia medica of ophthalmology and then, under the title of repertory, reviews the clinical manifestations.

---

**Text Book of Histology.** Frederick R. Bailey, A. M., M. D., published by Wm. Wood & Co., N. Y. 5th revised edition, 652 pages, 392 illustrations, $3.75.

This work is divided into parts as follows: Histologic Technic, the Cell, the Tissues, the Organs. Classifications of systems and apparatus appear mainly under the last heading and it will be noted that the general order automatically reviews and impresses on the mind, preliminary considerations. While the work is by no means abridged, each chapter contains a list of references for further study. Technic is given briefly for each structure, as well as in general terms in the first part. It will be realized, therefore, that the work is not merely one of technical detail, appealing only to the histologist, but one of considerable educational value and general interest. It is scarcely necessary to state that the book is well written and accurate as a source of information.

---

**The Practice of Urology.** Charles H. Chetwood, M. D., LL.D., F. A. C. S., N. Y. 2d edition, 310 illustrations, 825 pages, $5.50, Wm. Wood & Co., N. Y.

Much attention is paid to endoscopy and Roentgenography but ordinary diagnostic methods, including urinary analysis, are thoroughly considered. The work includes venereal diseases, over 100 pages being devoted to syphilis. The frontispiece reproduces a series of moving picture films illustrating the operation of nephrectomy. It will be noted that the work is rather more extensive than the recent limited conception of urology. It also combines a consideration of the needs of the urologist, essaying major operations and diagnostic procedures, and those of the general practitioner.

---

**Annual Report of the Surgeon General of the Public Health Service of the U. S., 1916.** Government Printing Office, Washington.. 421 pages, cloth binding.

---

**Transactions of the American Surgical Association.** Vol. 34, edited by Dr. John F. Binnie, Recorder, Kansas City. Printed for the Assn. by Wm. J. Dornan, Philadelphia. 540 pages, cloth binding. Meeting of May 9-11, 1916.

---

**Transactions of the Section on Obstetrics, Gynaecology and Abdominal Surgery, of the A. M. A.**, at the 67th annual session, at Detroit, June 13-16, 1916. A. M. A. Press, Chicago. 364 pages, cloth binding.

---

**The Rockefeller Foundation, Annual Report, 1915.** 61 Broadway, N. Y. 377 pages, paper covers.

---

**Progressive Medicine,** Vol. 19, No. 4, Dec., 1916. Edited by Hobart Amory Hare and Leighton F. Appleman, Philadelphia. Published by Lea & Febiger, Philadelphia. 466 pages, paper covers. Quarterly, $6 per annum.

Contents: Diseases of the Digestive Tract, etc., E. H. Goodman; Kidneys, J. H. Austin; G. U. Diseases, C. W. Bonney; Various Surgical Topics, J. C. Bloodgood; Practical Therapeutic Referendum, H. R. M. Landis.

---

**Transactions of the 38th Annual Meeting of the American Laryngological Association,** Washington, May 9-11, 1916. Published by the Assn., Dr. Harmon Smith, N. Y., Secretary. 326 pages, cloth binding.

---

**The Mulford Digest,** Vol. 3, No. 3, Dec., 1916.

This issue commemorates the 25th anniversary of the Mulford Laboratories in and near Philadelphia, is copiously illustrated and deals mainly with serum and bacterin therapy, vaccines and the like. Such work as is here described, bridges the gap between scientific theory and humanitarian results and should be studied by the medical profession. We may further emphasize that only the narrowest view of ethics prevents a full and cordial acknowledgement by the medical profession of the enormous debt of gratitude that they, as representatives of the health of the people, owe to this type of conservative, yet progressive research and constructive work.

---

**The Mentally Defective Child.** Meredith Young, M. D., D. P. H., D. S. Sc., Manchester, Eng., published by Paul B. Hoeber, 69 E. 59, N. Y. 137 pages, $1.50.

The author, being a barrister-at-law, is able to view this problem from a double standpoint. The work is especially prepared for teachers and enters somewhat into physical stigmata the anatomic and physiologic basis of mentality, discipline and school sanitation and hygiene. One of the best sentences in the book contains this statement: "......Some of us who are not good at paraphrasing, might run a serious risk of being classed as mentally defective"—referring to tests along the line of somewhat abstruse or extremely trite and obvious questions on ethical points. A number of illustrative cases are given.

---

**The Practical Medicine Series.** Charles L. Mix, A. M., M. D., general editor. The Year Book Publishers, 327 S. La Salle St,. Chicago. 10 volumes in annual series, $10.

Vol. 6, General Medicine, Frank Billings, M. S., M. D., and Burrell O. Raulston, A. B., M. D., editors, 342 pages, $1.50 separately.

Vol. 7, Obstetrics edited by Joseph B. De Lee, A. M., M. D., and Herbert M. Stowe, M. D., editors, 228 pages, $1.35 separately.

Vol. 8, Materia Medica and Therapeutics, edited by Geo. F. Butler, Ph. G., A. M., M. D. Preventive Medicine, edited by Wm. A. Evans, M. S., M. D., LL.D., Ph. D., 328 pages, $1.50 separately.

This review of current literature has been carried on for over 10 years, in a highly satisfactory manner. The division of the literature into sections, medicine and surgery usually occupying 2 volumes each, and the grouping of smaller sections with others, offers a convenient and economic choice to those not desiring to cover the whole field of medicine and also facilitates the assembling of the notes. The excerpts are sufficiently full of references as they stand, without requiring consultation of the original sources, unless for special purposes and illustrations are frequently reproduced.

